# Comparison of bipolar plasmakinetic resection of prostate versus photoselective vaporization of prostate by a three year retrospective observational study

**DOI:** 10.1038/s41598-021-89623-4

**Published:** 2021-05-12

**Authors:** Xu Cheng, Chuying Qin, Peng Xu, Yijian Li, Mou Peng, Shuiqing Wu, Da Ren, Lizhi Zhou, Yinhuai Wang

**Affiliations:** Department of Urology, The Second Xiangya Hospital of Central South University, Renmin Middle Road 139, Changsha, 410011 China

**Keywords:** Health care, Signs and symptoms, Urology

## Abstract

Comprehensive evaluation of photoselective vaporization of the prostate (PVP) versus plasmakinetic resection of the prostate (PKRP) in treating benign prostatic hyperplasia (BPH) is inadequate. This single-centre, retrospective observational study was designed to compare their efficacy, complications and sexual function. A total of 215 patients under PVP or PKRP were included in the study, propensity score matching (PSM) was performed to match the baseline characteristics of the two groups, and perioperative and three-year follow-up data were compared between them. Finally, 120 patients (60 for PVP and 60 for PKRP) were matched after PSM. Compared with the PKRP group, the intraoperative haemoglobin loss was lower (9.08 vs 13.75 g/L, P < 0.001) and the duration of catheterization and postoperative hospital stay were shorter (2.97 vs 4.10 day, P < 0.001; 3.95 vs 5.13 day, P < 0.001, respectively), but the operation time was longer (56.72 vs 49, 90 min, P < 0.001) in the PVP group. Urination measurements were improved for both groups after surgery, although no significant differences were found between them during follow-up. Sexual function after surgery was partly increased; however, frequent retrograde and discomfortable ejaculation occurred in both groups. In addition, dysuria incidence and retreatment were higher in the PVP group at 12 months. In conclusion, PVP is safe and effective in relieving BPH-related lower urinary tract symptoms with less perioperative blood loss and earlier recovery without inferior sexual function effects. However, the study is potentially affected by residual unmeasured confounding.

## Introduction

Current treatment options for benign prostatic hyperplasia (BPH)-related lower urinary tract symptoms (LUTSs) include observation, drug therapy and surgical intervention. At present, monopolar transurethral resection of the prostate (TURP) is still considered the "gold-standard" operation for treating BPH-related LUTS^1, 2^. In recent years, 160 W photoselective vaporization of the prostate (PVP) and bipolar plasmakinetic resection of the prostate (PKRP) have been widely used. The efficacy of PKRP is similar to that of TURP, while perioperative complications are greatly reduced^3–5^. The curative effect of PVP is similar to that of TURP, it has a good haemostasis effect, and its surgical-related complications are fewer than those of TURP^6–8^. Both PKRP and PVP may replace monopolar TURP as the gold standard of surgical treatment for BPH^9–11^.

However, the present studies comparing PVP and PKRP reported controversial results in some respects; moreover, few studies have investigated sexual function effects in the same cohort and with adequate follow-up. Therefore, we performed a retrospective, propensity score-matched (PSM) study with a 3-year follow-up to comprehensively compare the efficacy, complications, and sexual function outcomes between PVP and PKRP.

## Methods

### Patients

Patients who underwent surgical treatment for BPH between January 2014 and August 2016 at the Second Xiangya Hospital of Central South University were retrospectively analysed. This study was approved by the ethics committee of the Second Xiangya Hospital of Central South University, and informed consent was obtained from all included participants.

The inclusion criteria were as follows: (1) age > 50 years old; (2) moderate to severe LUTSs that seriously affect the quality of life of patients, an International Prostate Symptom Score (IPSS) > 7 points, and a maximal urinary flow rate (Qmax) < 15 ml/s; (3) drug treatment failure; (4) prostate volume < 100 g; and (5) an IPSS and quality of life (QoL) score that could be completed independently and effectively.

The exclusion criteria were as follows: (1) previous prostate or transurethral surgery; (2) total prostate specific antigen (tPSA) > 10 ng/ml or rectal finger examination showing prostate induration preoperatively and a diagnosis of prostate cancer on the basis of transrectal prostate biopsy; (3) the deterioration of comorbid medical diseases or a history of acute attack in the last 3 months; (4) urodynamic examination could not exclude neurogenic bladder or detrusor weakness; and (5) other suspected tumours or other diseases requiring surgical treatment, such as indirect inguinal hernia, bladder tumours and bladder stones.

### Study design

The study was a single-centre, retrospective, PSM study comparing 160-week PVP and PKRP with a 3-year follow-up. The primary assessment included baseline characteristics, as shown in Table 1. The operation time, haemoglobin loss during operation, duration of catheterization and hospital stay after operation were counted.

**Table 1:** Baseline patient characteristics.

Characteristic	Before PSM			After PSM		
	PVP group (n = 109)	PKRP group (n = 106)	P Value	PVP group (n = 60)	PKRP group (n = 60)	P Value
Age (year)	72.68 ± 6.95	67.47 ± 7.39	< 0.001	70.37 ± 6.09	71.12 ± 6.07	0.500
BMI (kg/m^2^)	21.93 ± 3.23	21.30 ± 3.16	0.146	21.68 ± 3.25	21.13 ± 3.12	0.341
PV (g)	63.19 ± 19.94	54.60 ± 21.00	0.002	59.40 ± 21.38	60.02 ± 20.33	0.872
tPSA (ng/ml)	5.18 ± 3.15	3.43 ± 2.48	< 0.001	4.07 ± 2.56	4.15 ± 2.64	0.856
IPSS	23.13 ± 3.80	21.74 ± 3.69	0.007	21.98 ± 4.30	22.92 ± 2.98	0.169
QoL	4.93 ± 0.65	4.79 ± 0.67	0.138	4.77 ± 0.70	4.97 ± 0.52	0.078
Qmax (ml/s)	6.63 ± 2.27	7.31 ± 2.40	0.034	7.24 ± 2.43	6.70 ± 1.84	0.176
PVR (ml)	84.06 ± 60.80	72.18 ± 59.87	0.150	72.32 ± 62.27	80.40 ± 55.78	0.455
IIEF-5	8.04 ± 1.67	8.96 ± 2.11	< 0.001	8.37 ± 1.79	8.27 ± 2.11	0.780
MSHQ	41.61 ± 18.98	48.38 ± 15.91	0.005	46.07 ± 17.59	43.10 ± 16.90	0.348
MSHQ-EjD	12.08 ± 5.02	14.16 ± 5.48	0.004	12.87 ± 5.21	12.00 ± 5.18	0.362
Charlson CI			0.035			0.678
0	42	49		27	22	
1	37	42		18	26	
2	21	14		9	12	
3	9	1		6	0	

*BMI* body mass index, *PV* prostate volume, *tPSA* total prostate-specific antigen, *IPSS* international prostate symptom score, *QoL* quality of life score, *Qmax* maximal urinary flow rate, *PVR* postvoid residual, *IIEF-5* international index of erectile function-five term score, *MSHQ* male sexual health questionnaire score, *MSHQ-EjD* male sexual health questionnaire on ejaculatory dysfunction score, *Charlson CI* charlson comorbidity index.

**Figure 1:**
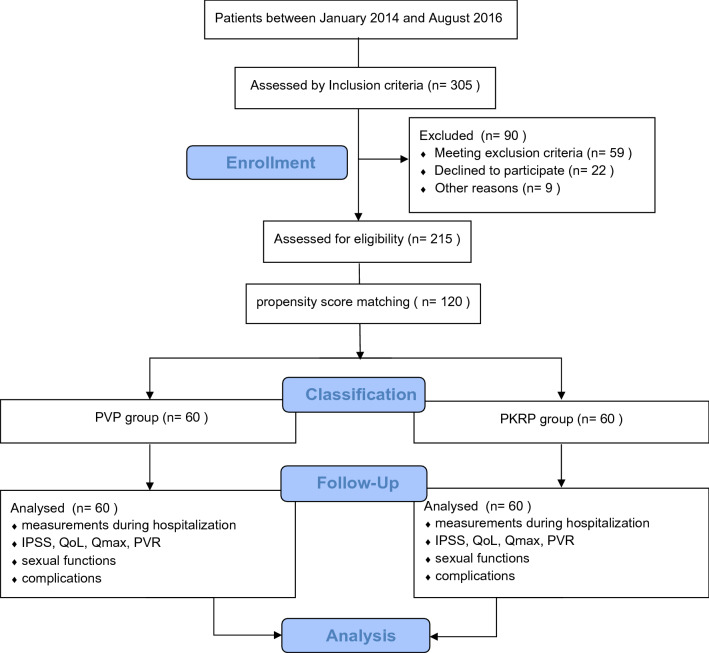
Flow diagram depicting cohort formation, treatment and follow-up of patients.

Postoperative follow-up data of 12 months, 24 months, and 36 months included three units: (1) efficacy measures: International Prostate Symptom Score (IPSS)^12^, maximal urinary flow rate (Qmax), quality of life (QoL) score^12^, and postvoid residual (PVR); (2) sexual function outcomes: International Index of Erectile Function-five term Score (IIEF-5)^13^, Male Sexual Health Questionnaire total Score (MSHQ) and Male Sexual Health Questionnaire-Ejaculatory Dysfunction Score (MSHQ-EjD)^14^, retrograde ejaculation and discomfort on ejaculation events; and (3) postoperative adverse events by Clavien-Dindo grading. This study was reported according to the Strengthening the Reporting of Observational Studies in Epidemiology guidelines for cohort studies^15^. The study is depicted by flow diagram as shown in Fig. 1.

### Surgical procedures

All included patients underwent surgery by one fully trained urologist. Exact procedures are described in the Supplementary Information.

### Propensity score-matching and statistical analysis

To eliminate significant differences in comparison of baseline characteristics, we calculated a propensity score using multivariate logistic models on the basis of those clinical factors that were significantly different in the whole cohort. We performed one-to-one greedy nearest-neighbour matching within one-quarter of the standard deviation of the estimated propensity to create comparable cohorts (caliper 0.02). Patients receiving PVP were then matched with patients receiving PKRP.

The figures were made by prism (version 8.0.1). All data were analysed by SPSS software (IBM, New York, NY, USA). The continuous variables are described as the mean ± standard deviation ($\overline{X }\pm SD$). The two-sample t test for continuous variables and Fisher’s exact test or chi-square test for categorical variables were used. Considering the matched nature of the dataset, paired t-test and McNemar test was also used for outcomes to check the consistency of test decisions (Supplementary Information). The difference was statistically significant when P < 0.05.

### Ethical statement

The study was conducted in accordance with the Declaration of Helsinki (as revised in 2013). The study was approved by the ethics committees at the Second Xiangya Hospital of Central South University. Informed consent was taken from all individual participants.

### Reporting guideline

This study was reported according to the Strengthening the Reporting of Observational Studies in Epidemiology guidelines for cohort studies.

## Result

### Baseline patient characteristics

The whole cohort consisted of 215 patients, with 109 patients receiving PVP and 106 patients receiving PKRP. As Table 1 shows, baseline patient characteristics for both arms were not comparable for the following clinical variables: age (72.68 vs 67.47 years, *P* < 0.001), prostate volume (63.19 vs 54.60 g, *P* = 0.002), tPSA (5.18 vs 3.43 ng/ml, *P* < 0.001), IPSS (23.13 vs 21.74, *P* = 0.007), Qmax (6.63 vs 7.31 ml/s, *P* = 0.03), IIEF-5 (8.04 vs 8.96, *P* < 0.001), MSHQ (41.16 vs 48.38, *P* = 0.005), MSHQ-EjD (12.08 vs 14.16, *P* = 0.004), and Charlson Comorbidity Index (42 vs 49, 37 vs 42, 21 vs 14, 9 vs 1, respectively, *P* = 0.04). After PSM on the basis of these variables, 60 patients for PVP and 60 patients for PKRP were matched for further investigation. There was no significant difference in baseline patient characteristics after PSM (Table 1).

### Intraoperative indicators

A longer operation time was required in the PVP group than in the PKRP group (56.72 vs 49, 90 min, *P* < 0.001). However, the haemoglobin loss in the PVP group was lower than that in the PKRP group (9.08 vs 13.75 g/l, *P* < 0.001), the catheterization time in the PVP group was shorter (2.97 vs 4.10 days, *P* < 0.001), and the duration of hospital stay was shorter in the PVP group than in the PKRP group (3.95 vs 5.13 days, *P* < 0.001).

### Postoperative comparison

**Figure 2:**
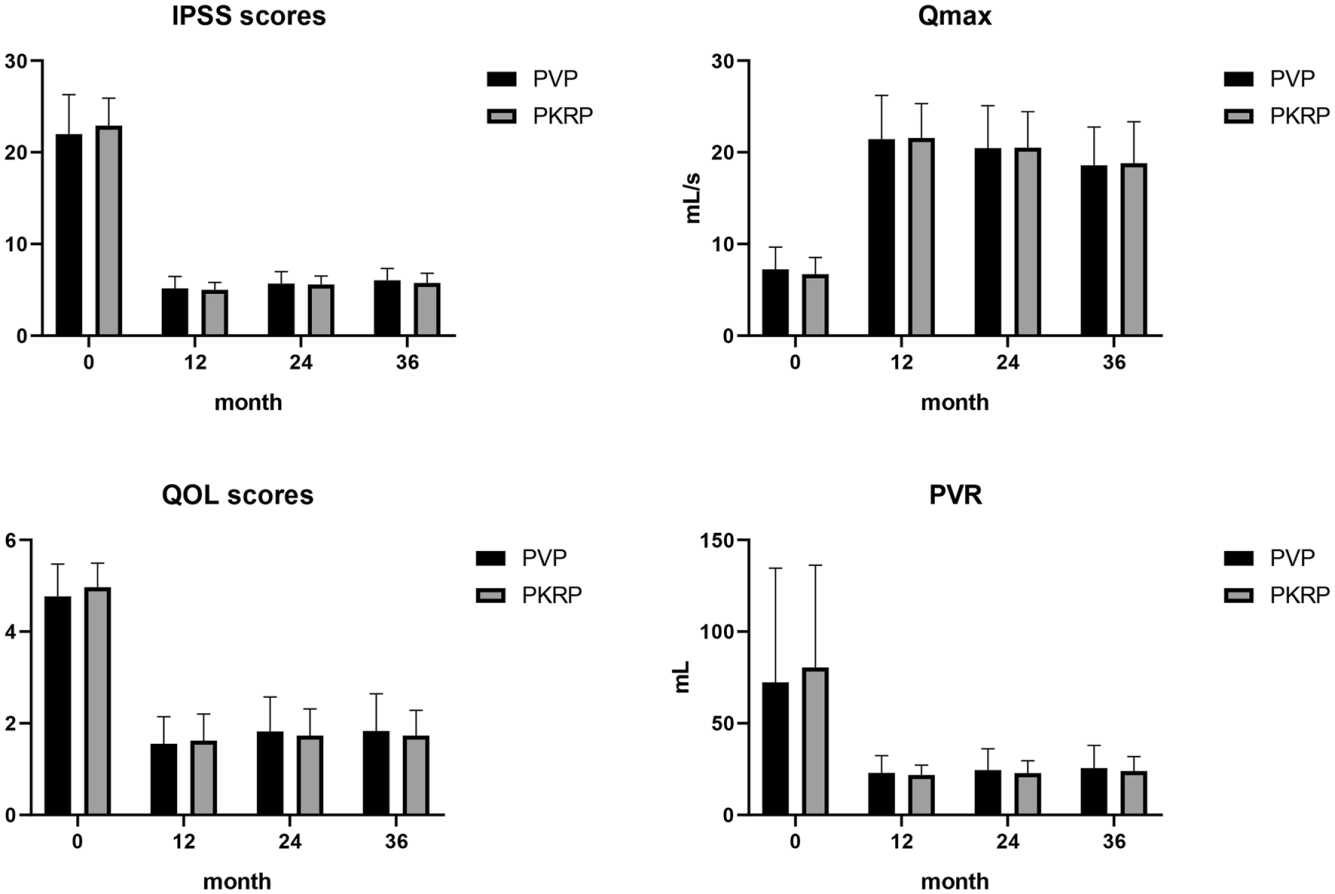
IPSS, QoL score, Qmax, and PVR during the 12-month, 24-month, and 36-month follow-up. (P < 0.05) (‘0’ represents preoperative).

No patients were lost during the follow-up period. A total of 120 patients were followed up for 36 months, including 60 patients in the PVP group and 60 patients in the PKRP group. During the 36-month follow-up, the Qmax and QoL of both groups significantly increased, and the IPSS and PVR significantly decreased compared with the preoperative level (Fig. 2). However, there were no significant differences in Qmax (21.45 *vs* 21.57 ml/s, 20.47 vs 20.52 ml/s, 21.45 vs 21.57 ml/s, *P* = 0.89;* P* = 0.95;* P* = 0.77, respectively), PVR (22.88 vs 21.83 ml, 24.38 vs 22.78 ml, 25.57 vs 23.88 ml, *P* = 0.45;* P* = 0.36;* P* = 0.38, respectively), IPSS (5.27 vs 5.00, 5.68 vs 5.60, 6.05 vs 5.75, *P* = 0.40; *P* = 0.69; *P* = 0.17, respectively) or QoL (1.55 vs 1.62, 1.82 vs 1.73, 1.83 vs 1.73, *P* = 0.54; *P* = 0.50; *P* = 0.43, respectively) between the PVP group and PKRP group at the 12-, 24-, and the 36-month follow-ups (Table 2).

**Table 2:** 3-year follow-up results of PVP group and PKRP group.

	12 months			24 months			36 months		
	PVP (n = 60)	PKRP (n = 60)	*P* value	PVP (n = 60)	PKRP (n = 60)	*P* value	PVP (n = 60)	PKRP (n = 60)	*P* value
IPSS	5.27 ± 1.28	5.00 ± 0.82	0.397	5.68 ± 1.31	5.60 ± 0.91	0.686	6.05 ± 1.29	5.75 ± 1.07	0.169
QoL	1.55 ± 0.59	1.62 ± 0.58	0.537	1.82 ± 0.75	1.73 ± 0.58	0.496	1.83 ± 0.81	1.73 ± 0.55	0.428
Qmax (ml/s)	21.45 ± 4.75	21.57 ± 3.74	0.881	20.47 ± 4.63	20.52 ± 3.91	0.949	18.58 ± 4.19	18.82 ± 4.52	0.770
PVR (ml)	22.88 ± 9.39	21.83 ± 5.30	0.452	24.38 ± 7.70	22.78 ± 6.80	0.362	25.57 ± 12.38	23.88 ± 7.92	0.377

*IPSS* International Prostate Symptom score, *QoL* Quality of Life score, *Qmax* maximal urinary flow rate, *PVR* postvoid residual.

**Figure 3:**
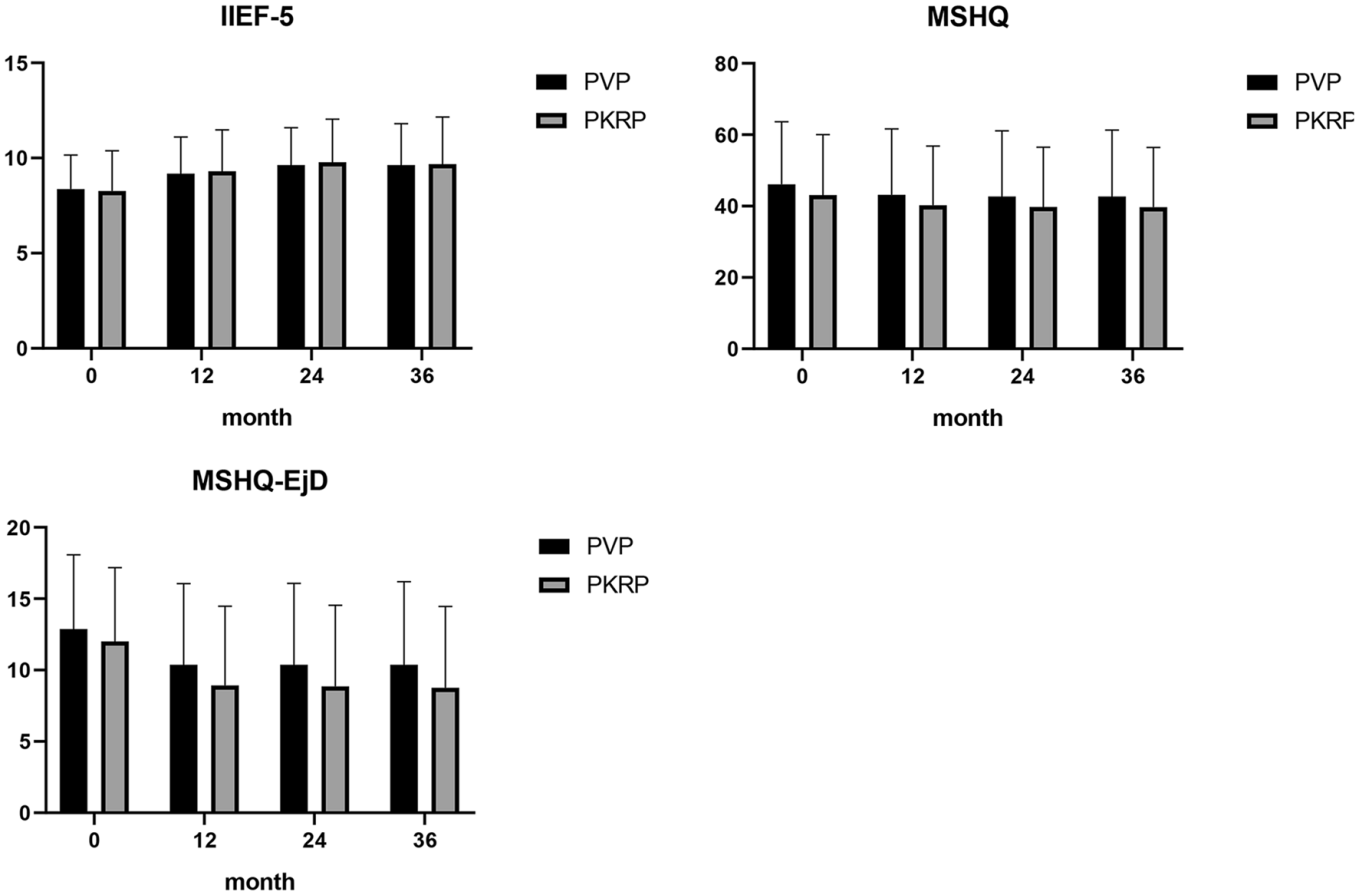
IIEF-5, MSHQ score, MSHQ-EjD score during the 12-month, 24-month, and 36-month follow-up. (P < 0.05) (‘0’ represents preoperative).

For sexual function outcomes, there was an increase in the IIEF-5 score of both groups, which suggested an erectile function improvement after surgery (Fig. 3). However, the postoperative MSHQ total score and MSHQ-EjD score were significantly decreased in both groups (Fig. 3). In addition, there were no significant differences in IIEF-5 (9.18 vs 9.30, 9.63 vs 9.78, 9.63 vs 9.68, *P* = 0.76; *P* = 0.70; *P* = 0.91, respectively), MSHQ (43.15 vs 40.20, 42.70 vs 39.78, 42.70 vs 39.68, *P* = 0.36; *P* = 0.37; *P* = 0.35, respectively), MSHQ-EjD (10.38 vs 8.93, 10.38 vs 8.85, 10.38 vs 8.75, *P* = 0.16; *P* = 0.14; *P* = 0.12, respectively), retrograde ejaculation (43 vs 44%, 40 vs 39%, 37 vs 37%, *P* = 0.84; *P* = 0.85; *P* = 1.00, respectively) or discomfortable ejaculation incidence (43 vs 47%, 38 vs 42%, 35 vs 35%, *P* = 0.52; *P* = 0.44; *P* = 1.00, respectively) between the PVP group and the PKRP group at the 12-, 24, and 36-month follow-ups (Table 3).

**Table 3:** 3-year follow-up results of sexual function in two groups.

	12 months			24 months			36 months		
	PVP (n = 60)	PKRP (n = 60)	*P* value	PVP (n = 60)	PKRP (n = 60)	*P* value	PVP (n = 60)	PKRP (n = 60)	*P* value
IIEF-5	9.18 ± 1.93	9.30 ± 2.18	0.757	9.63 ± 1.96	9.78 ± 2.26	0.698	9.63 ± 2.18	9.68 ± 2.47	0.907
DOE (%)	44 (73.33)	47 (78.33)	0.522	38 (63.33)	42 (70.00)	0.439	35 (58.33)	35 (58.33)	1.000
RE (%)	43 (71.67)	44 (73.33)	0.838	40 (66.67)	39 (65.00)	0.847	37 (61.67)	37 (61.67)	1.000
MSHQ-EjD	10.38 ± 5.67	8.93 ± 5.54	0.159	10.38 ± 5.69	8.85 ± 5.69	0.142	10.38 ± 5.81	8.75 ± 5.71	0.123
MSHQ	43.15 ± 18.47	40.20 ± 16.66	0.360	42.70 ± 18.42	39.78 ± 16.74	0.366	42.70 ± 18.61	39.68 ± 16.75	0.352

*DOE* discomfort on ejaculation, *RE* retrograde ejaculation, *IIEF-5* international index of erectile function-five term score, *MSHQ* male sexual health questionnaire score, *MSHQ-EjD* male sexual health questionnaire on ejaculatory dysfunction score.

All the patients in the two groups underwent surgery successfully. The complication data classified using the Clavien-Dindo scale are shown in Table 4. At the 12-month follow-up, dysuria incidence was higher in the PVP group (8 vs 2% of Grade II, *P* < 0.05; 10 vs 1% of Grade III, *P* = 0.001). There were no differences in other complications between the two groups. Furthermore, retreatment at 12 months (regardless of the cause) occurred more frequently in the PVP patients (12 vs 3%, *P* = 0.01).

**Table 4:** Adverse events in each study period.

		0–12 months			12–24 months			24–36 months		
		PVP (n = 60)	PKRP (n = 60)	*P* value	PVP (n = 60)	PKRP (n = 60)	*P* value	PVP (n = 60)	PKRP (n = 60)	*P* value
Clavien-Dindo Grade I	IS (%)	16	16		5	5		0	0	
	UTI (%)	3	6		0	0		0	0	
	UI (%)	3	3		0	0		0	0	
	UR (%)	4	3		0	0		0	0	
Clavien-Dindo Grade II	DU (%)	8	2	0.048	0	0		0	0	
	IS (%)	6	4		0	0		0	0	
	UI (%)	2	3		0	0		0	0	
	US (%)	1	2		0	0		0	0	
Clavien-Dindo Grade III	US (%)	2	4		0	0		0	0	
	UI (%)	1	0		0	0		0	0	
	DU (%)	10	1	0.004	0	0		0	0	
	UR (%)	4	4		2	0		0	0	
All cause retreatment (%)		12	3	0.013	14	6	0.050	13	9	0.345

*Clavien-Dindo Grade* the Clavien-Dindo classification of surgical complications into 5 grades (I, II, III, IV and V), *IS* irritative symptoms, *US* Urethral stricture, *UI* urinary incontinence, *UTI* urinary tract infection, *UR* urinary retention, *DU* Dysuria.

## Discussion

BPH-related LUTS is one of the most common conditions in elderly men and seriously affects their health and quality of life. Surgical intervention is the most effective treatment for BPH. Monopolar TURP has been regarded as the "gold standard" of BPH surgery for nearly half a century. With the development of medical equipment, PKRP and PVP are becoming alternatives to monopolar TURP. Some studies have shown that the efficacy of PKRP or PVP is similar to that of TURP, but the incidence of related complications is much lower than that of TURP^6, 16–18^. Liu reported that PKRP was superior to TURP in terms of serum sodium reduction, hospital stay, blood transfusion rate, TURS and urethral stricture incidence, while there was no significant difference in IIEF-5 or retrograde ejaculation^19^. Many studies have compared PVP with PKRP, indicating their efficacy and safety in the treatment of BPH^20–25^. However, studies comprehensively comparing their effects with adequate follow-up are lacking.

In the present study, we found that the intraoperative blood loss in the PVP group was significantly less than that in the PKRP group, which may be due to the good haemostasis performance of green light. The average operation time in the PVP group was clearly longer, but when using a 160 W high-power laser, this small difference is not clinically relevant. During the whole follow-up, we found no significant differences in IPSS, QoL, Qmax, PVR or sexual function outcomes between the two groups, which is consistent with most published studies^2, 26, 27^. Patients in the two groups had improved postoperative erectile function as a result of LUTS relief and a better QoL. However, the discomfort and retrograde ejaculation incidence at 12 months was high for both PVP and PKRP, which is comparable to that previously reported for monopolar TURP^27^. The exact mechanism of ejaculation has not been well defined, and the structure of the bladder neck and ejaculatory hood are necessary for antegrade ejaculation^28, 29^; thus, surgical strategies preserving these structures are worth exploring.

On the other hand, this study suggested that perioperative complications are comparable between PVP and PKRP, but we did find that early dysuria incidence was higher in the PVP group. In this study, we defined dysuria as pain and/or burning, stinging, or itching of the urethra or urethral meatus with urination. There are many causes of dysuria. Here, we focus on surgical-procedure-related, noninfectious inflammatory causes, such as urethral anatomic abnormalities and local trauma. The higher dysuria rate in PVP may be because of thermal damage and oedema in urethral tissue in the early stage. In addition, the all-cause-retreatment rate is slightly higher in PVP patients, and the reason can be dysuria-related or inadequate laser energy delivery, leading to incomplete tissue removal; the latter would be markedly solved with 180 W laser systems.

This study still has shortcomings: it is a single-centre retrospective observational cohort study analysed by PSM, and is potentially affected by residual unmeasured confounding. Even so, we can also conclude that compared with PKRP, PVP is a safe and effective choice to relieve BPH-related lower urinary tract symptoms without inferior sexual function effects, and PVP is better in perioperative blood loss and early recovery after surgery. However, surgical strategies preserving more functions, including ejaculation, are still worth exploring.

## Supplementary Information

Supplementary Information 1.

Supplementary Information 2.

## Data Availability

The datasets generated during and analyzed during the current study are available from the corresponding author on reasonable request.
