# Supplementary material for: Comparison of bipolar plasmakinetic resection of prostate versus photoselective vaporization of prostate by a three year retrospective observational study
Source: Sci Rep. 2021 May 12;11:10142. doi: 10.1038/s41598-021-89623-4 (PMC8115102; doi:10.1038/s41598-021-89623-4)
Supplement: Supplementary file 1 — Supplementary Information 1. [file 41598_2021_89623_MOESM1_ESM.docx]

**Title:** Comparison of bipolar plasmakinetic resection of prostate versus photoselective vaporization of prostate by a three year retrospective observational study

**Authors:** Xu Cheng, Chuying Qin, Peng Xu, Yijian Li, Mou Peng, Shuiqing Wu, Da Ren, Lizhi Zhou, Yinhuai Wang

## Supplementary materials

***Surgical Procedures***

Patients were given general anesthesia/epidural anesthesia/ spinal anesthesia.

PVP group: All patients in this group were operated by the same experienced surgeon. The operation uses the non-contact direct green laser treatment system of the Realton company, the 23F urethra cystoscope and the Storz HD camera system of Germany Storz company, and the straight-out optical fiber, with normal saline as the lavage fluid. The green laser vaporization output power 160W, the hemostatic output power 40W. After general anesthesia, bladder lithotomy was taken. 23F urethral cystoscope was placed through urethra under direct vision. The operation was terminated after establishing a smooth, non-obstructive prostatic urethra. After observation of nonactive hemorrhage, 200 ml saline was injected into the bladder to fill the bladder and withdraw from cystoscopy, press the suprapubic area lightly and coarse urinary flow was seen from the external urethral orifice. Three chambers Foley catheter(F22 type) was retained. 40 ml saline was injected into the balloon. No need of routine traction and fixation of the catheter or continuous bladder irrigation.

PKRP group: All patients in this group were operated on by the same experienced surgeon. The operation was performed by a bipolar plasmakinetic system. Saline was used as the lavage fluid. The output power was 280W and the hemostatic output power was 60W. The gland tissues were washed out with Elik irrigator and sent for histopathological examination. Traction and fixation of the catheter and continuous bladder irrigation were performed routinely after operation.
