# Supplementary material for: Comparison of bipolar plasmakinetic resection of prostate versus photoselective vaporization of prostate by a three year retrospective observational study
Source: Sci Rep. 2021 May 12;11:10142. doi: 10.1038/s41598-021-89623-4 (PMC8115102; doi:10.1038/s41598-021-89623-4)
Supplement: Supplementary file 2 — Supplementary Information 2. [file 41598_2021_89623_MOESM2_ESM.docx]

**Title:** Comparison of bipolar plasmakinetic resection of prostate versus photoselective vaporization of prostate by a three year retrospective observational study

**Authors:** Xu Cheng, Chuying Qin, Peng Xu, Yijian Li, Mou Peng, Shuiqing Wu, Da Ren, Lizhi Zhou, Yinhuai Wang

## Supplementary materials

***Paired t-test and McNemar test for main outcomes***

|  | t-test | paired t-test | Chi squared test | McNemar test |
| --- | --- | --- | --- | --- |
| hemoglobin loss | p < 0.001 | p < 0.001 |  |  |
| 12-month IPSS | p=0.397 | p=0.396 |  |  |
| 36-month retreatment |  |  | p=0.345 | p=0.481 |

***Datasets***

| PVP | PKRP | frequency |
| --- | --- | --- |
| 1 | 1 | 2 |
| 1 | 0 | 11 |
| 0 | 1 | 7 |
| 0 | 0 | 40 |

36-month retreatment; 1=retreatment; 0=non-retreatment

| blood loss_PVP(mL) | blood loss_PKRP(mL) | ipss12_PVP | ipss12_PKRP |
| --- | --- | --- | --- |
| 21 | 23 | 4 | 5 |
| 14 | 18 | 4 | 4 |
| 14 | 12 | 4 | 5 |
| 17 | 13 | 4 | 6 |
| 12 | 14 | 5 | 7 |
| 15 | 2 | 5 | 5 |
| 15 | 9 | 5 | 4 |
| 11 | 11 | 5 | 4 |
| 12 | 7 | 5 | 5 |
| 9 | 6 | 5 | 7 |
| 13 | 17 | 5 | 5 |
| 11 | 9 | 5 | 6 |
| 13 | 6 | 5 | 6 |
| 8 | 14 | 5 | 5 |
| 8 | 17 | 5 | 4 |
| 4 | 18 | 6 | 3 |
| 5 | 17 | 6 | 5 |
| 0 | 30 | 6 | 7 |
| 0 | 7 | 7 | 6 |
| 0 | 14 | 10 | 5 |
| 2 | 7 | 6 | 4 |
| 6 | 17 | 5 | 5 |
| 15 | 3 | 4 | 4 |
| 6 | 26 | 4 | 5 |
| 15 | 11 | 3 | 4 |
| 5 | 16 | 5 | 5 |
| 5 | 2 | 4 | 5 |
| 11 | 16 | 5 | 4 |
| 9 | 31 | 5 | 4 |
| 6 | 29 | 5 | 5 |
| 28 | 16 | 4 | 6 |
| 2 | 26 | 6 | 4 |
| 14 | 14 | 6 | 4 |
| 9 | 46 | 6 | 5 |
| 4 | 27 | 7 | 4 |
| 2 | 24 | 5 | 4 |
| 3 | 22 | 6 | 4 |
| 10 | 12 | 4 | 5 |
| 0 | 12 | 6 | 5 |
| 16 | 12 | 5 | 5 |
| 15 | 12 | 6 | 5 |
| 6 | 12 | 6 | 5 |
| 5 | 11 | 5 | 5 |
| 7 | 11 | 4 | 5 |
| 13 | 10 | 5 | 5 |
| 4 | 10 | 5 | 5 |
| 7 | 10 | 3 | 5 |
| 23 | 10 | 4 | 5 |
| 4 | 10 | 3 | 5 |
| 14 | 10 | 6 | 5 |
| 3 | 10 | 5 | 5 |
| 13 | 10 | 5 | 5 |
| 12 | 9 | 4 | 5 |
| 8 | 9 | 10 | 5 |
| 10 | 9 | 4 | 5 |
| 4 | 9 | 5 | 6 |
| 0 | 8 | 6 | 6 |
| 11 | 8 | 6 | 6 |
| 1 | 7 | 6 | 6 |
| 15 | 7 | 5 | 6 |

Processed by SPSS software (version 22.0)
